# Exploring Heavy Metals Exposure in Urban Green Zones of Thessaloniki (Northern Greece): Risks to Soil and People’s Health

**DOI:** 10.3390/toxics13080632

**Published:** 2025-07-27

**Authors:** Ioannis Papadopoulos, Evangelia E. Golia, Ourania-Despoina Kantzou, Sotiria G. Papadimou, Anna Bourliva

**Affiliations:** 1 Soil Science Laboratory, School of Agriculture, Faculty of Agriculture, Forestry and Natural Environment, Aristotle University of Thessaloniki, University Campus, 541 24 Thessaloniki, Greece; papadopoulosjohn28@gmail.com (I.P.); egolia@agro.auth.gr (E.E.G.); kantzourania@gmail.com (O.-D.K.); sotiriapg@agro.auth.gr (S.G.P.); 2 Institute of Soil and Water Resources, Hellenic Agricultural Organization DIMITRA (ELGO Dimitra), 570 01 Thessaloniki, Greece; 3 Department of Agriculture, Crop Production and Rural Environment, University of Thessaly, 384 46 Volos, Greece

**Keywords:** urban soils, heavy metals, source apportionment, contamination indices, health risk

## Abstract

This study investigates the heavy metal contamination in urban and peri-urban soils of Thessaloniki, Greece, over a two-year period (2023–2024). A total of 208 composite soil samples were systematically collected from 52 sites representing diverse land uses, including high-traffic roadsides, industrial zones, residential neighborhoods, parks, and mixed-use areas, with sampling conducted both after the wet (winter) and dry (summer) seasons. Soil physicochemical properties (pH, electrical conductivity, texture, organic matter, and calcium carbonate content) were analyzed alongside the concentrations of heavy metals such as Cd, Co, Cr, Cu, Mn, Ni, Pb, and Zn. A pollution assessment employed the Geoaccumulation Index (I_geo_), Contamination Factor (Cf), Pollution Load Index (PLI), and Potential Ecological Risk Index (RI), revealing variable contamination levels across the city, with certain hotspots exhibiting a considerable to very high ecological risk. Multivariate statistical analyses (PCA and HCA) identified distinct anthropogenic and geogenic sources of heavy metals. Health risk assessments, based on USEPA models, evaluated non-carcinogenic and carcinogenic risks for both adults and children via ingestion and dermal contact pathways. The results indicate that while most sites present low to moderate health risks, specific locations, particularly near major transport and industrial areas, pose elevated risks, especially for children. The findings underscore the need for targeted monitoring and remediation strategies to mitigate the ecological and human health risks associated with urban soil pollution in Thessaloniki.

## 1. Introduction

The pollution of the living environment that humans occupy and develop every function of life in, including career development and life support, is a critical issue that impacts all areas of their existence [1]. Polluted air is well-known to directly affect human health, as due to inhalation, toxic pollutants can enter the body and generate a wide range of issues that threaten safe and healthy living [2]. Meanwhile, pollutants are also deposited on plant leaves, on the soil surface, and in the water bodies of the earth’s surface, which provide irrigation for crops and the living environment for every kind of organism [3]. The link between air, water, and soil is a reality, as pollutants can move, adsorb, diffuse, and become more or less available, posing a direct threat to human health [4].

Heavy metals are among the most dangerous and persistent pollutants in the environment and particularly in soils [5]. Heavy metals have been a vexing quandary for scientists involved in environmental studies for the last 50 years, trying to identify their possible sources, their chemical behavior, and possible mechanisms to reduce their concentration or availability [6]. The presence of heavy metals persists in both agricultural and urban soils due to a variety of human activities in farming practices or in urban green areas in numerous cities around the world [7].

Urban soils constitute a special and distinct soil class as they have diverse features since they are affected by anthropogenic interventions like buildings and roads [8]. Industrial installations are often located close to them, in areas classified as peri-urban. Moreover, it is not possible to present common and uniform characteristics, as the urban land surfaces are extremely small and discontinuous [9]. However, in the heart of cities, parks with playgrounds are needed where children and adults alike can enjoy their walks or daily exercise [10]. Schools serving the urban population are also being established and operating intensively, trying to meet the educational needs of children and adolescents aged 4 to 18 [11]. University campuses or colleges with courtyards where students spend many hours a day are located in many large cities [12]. Urban green spaces within cities are contaminated with a wide range of pollutants including heavy metals, which pose a potential risk to anyone walking, exercising, playing sports, or engaging in activities within them [13].

Scientists in Greece have carried out studies to assess pollution in numerous sites [14] in major cities [13], including the capital, Athens [15], and the next largest city, Thessaloniki [16,17]. Athens and Thessaloniki are located at different geographical altitudes, central Greece and northern Greece, respectively, where different climatic conditions prevail. In Thessaloniki, rainfall is more frequent, with high intensity and large volumes of water [18]. It has a coastal front, a commercial and passenger port with rich activity and increased traffic, especially in the summer months as there is a constant presence of cruise ships [19]. Located at the beginning of the coastal front and near the commercial port, there is a large railway station with high traffic as it connects Thessaloniki with the rest of Greece, as well as with Balkan countries and Central European countries [20]. West of the city, towards the same side of the city, lies a busy long-distance train station, with daily and all-day services in many different directions, as it allows Thessaloniki to be connected to cities in northern Greece, Macedonia, and Thrace. With the operation of the major highways, wheeled vehicles move at high speeds, especially following the COVID-19 period [21].

The objective of the current study is to investigate the heavy metal pollution of the city of Thessaloniki. The study was carried out using appropriate pollution indicators, highlighting the degradation risk and ecological risk. Finally, indices related to the health risk for Thessaloniki residents were also calculated, and their spatial distribution was highlighted by constructing corresponding maps.

## 2. Materials and Methods

### 2.1. Study Area and Sampling

**Figure 1:**
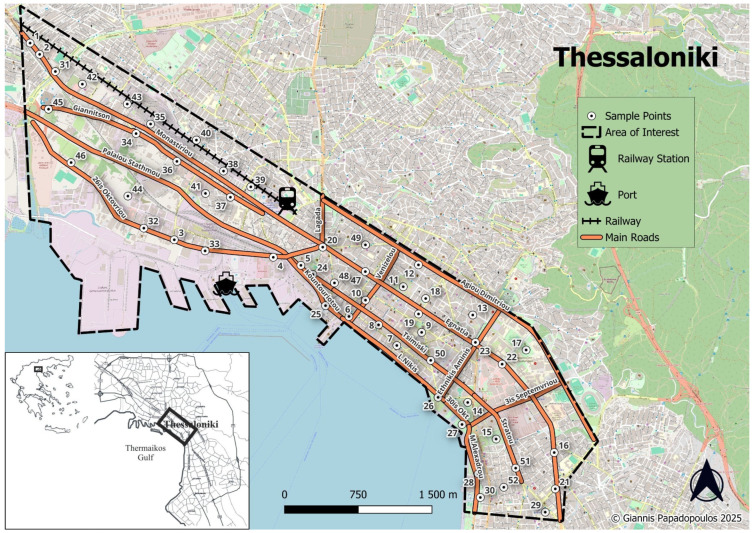
Study area and sampling points of urban soils in the city of Thessaloniki.

Soil sampling was carried out in the greater urban and peri-urban area of Thessaloniki over a two-year period from 2023 to 2024 to evaluate the seasonal and spatial distribution of heavy metals in urban soils as presented in Figure 1. A total of 208 composite soil samples (104 samples per year) were collected. Soil samples were repeatedly collected from 52 sampling sites strategically distributed across different land use types, including high-traffic roadsides, industrial zones, residential neighborhoods, parks, and mixed-use areas. Each year, soil sampling was conducted during two distinct periods: once in March, following the wet season (hereafter referred to as winter—W), and again in September, after the dry period characterized by high summer temperatures and just before the onset of autumn rainfall (hereafter referred to as summer—S), ensuring temporal consistency and capturing potential temporal variation in soil contamination. At each site, the envelope method was applied to enhance spatial representativeness. Specifically, five sub-samples were collected from the four corners and the center of a 1 m × 1 m square plot. These sub-samples were then thoroughly mixed in a clean plastic container to produce a single composite sample per site. A total of 1 kg of soil was collected from a depth of 0–10 cm using a special wooden sampler without metallic parts to avoid cross-contamination. Samples were placed in labeled polyethylene bags, stored under ambient conditions, and transported to the Laboratory of Soil Science of Aristotle University of Thessaloniki where all samples were air-dried at room temperature, disaggregated, and passed through a 2 mm sieve to remove debris and stones. The prepared samples were then stored in sealed containers until physicochemical analysis.

### 2.2. Soil Physicochemical Analyses

To characterize the basic properties influencing the mobility and bioavailability of heavy metals, a series of physicochemical analyses were performed on all soil samples. These analyses included soil pH, electrical conductivity (EC), and textural characterization, following standard laboratory protocols [22]. Specifically, soil pH was measured using a digital pH meter, while electrical conductivity (EC) was determined with a conductivity meter, both in an aqueous suspension at a soil-to-water ratio of 1:2.5 [23]. Soil texture was assessed by quantifying the proportions of sand, silt, and clay using the Bouyoucos method following the chemical dispersion of clay particles with sodium hexametaphosphate [24]. The concentrations of Cd, Co, Cr, Cu, Min, Ni, Pb, and Zn were measured using an Atomic Absorption Spectrophotometer (Shimadzu 6300). Prior to analysis, soil samples underwent extraction with a 3:1 mixture of concentrated hydrochloric and nitric acid using a heated, closed-vessel digestion system. The resulting extracts were then diluted to the appropriate volume and analyzed following the preparation of individual calibration curves for each metal. To ensure the accuracy of the measurements, a certified reference soil sample was analyzed alongside the test samples. The recovery rates of the method were found to range between 97.4% and 102.3%, confirming the reliability of the procedure.

### 2.3. Contamination Indices

To comprehensively assess the extent and potential impact of heavy metal contamination in urban soils, a suite of widely used pollution and risk assessment indices—namely the Geoaccumulation Index (I_geo_), Contamination Factor (C_f_), Pollution Load Index (PLI), and Potential Ecological Risk Index (RI)—were applied in this study and are described in detail below.

#### 2.3.1. Geoaccumulation Index (I_geo_)

The Geoaccumulation Index (I_geo_), originally proposed by Müller [25], was calculated using the following formula:

(1) $$I_{geo}={log}_{2}\left(\frac{C_{n}}{1.5\times B_{n}}\right)$$

In this equation, C_n_ represents the measured concentration of the element n in the urban soil samples, B_n_ denotes the geochemical background concentration (i.e., Upper Continental Crust—UCC values) of the same element, and 1.5 serves as a correction factor to account for natural variations in the background matrix caused by lithological differences. According to I_geo_ values, the urban soils are characterized as follows: I_geo_ ≤ 0 indicates uncontaminated, 0–1 uncontaminated to moderately contaminated, 1–2 moderately contaminated, 2–3 moderately to heavily contaminated, 3–4 heavily contaminated, 4–5 heavily to extremely contaminated, and I_geo_ > 5 indicates extremely contaminated soil samples.

#### 2.3.2. Contamination Factor (C_f_) and Pollution Load Index (PLI)

The Contamination Factor $C_{f}^{i}$, introduced by Hakanson [26], is calculated as follows:

(2) $$C_{f}^{i}=\frac{C_{n}^{i}}{B_{n}^{i}}$$

where $C_{f}^{i}$ is the concentration of a specific element in the urban soils, while $B_{n}^{i}$ is the geochemical background value of the same element. This factor serves as an indicator of the degree of anthropogenic pollution for each element, where higher values reflect greater contamination relative to natural background levels. According to classification criteria, a $C_{f}$ value less than 1 indicates low contamination, values between 1 and 3 denote moderate contamination, 3 to 6 suggest considerable contamination, and values above 6 reflect very high contamination [26].

The Pollution Load Index (PLI), a multi-elemental index proposed by Tomlinson et al. [27], is calculated as the nth root of the multiplication of the individual Contamination Factors $C_{f}^{i}$ for all analyzed elements. The formula is expressed as follows:

(3) $$PLI=\sqrt[{n}]{\left({Cf}_{1}\times {Cf}_{2}\times \ldots \times {Cf}_{n}\right)}$$

A PLI value greater than 1 indicates that the soil is contaminated, while a PLI value less than 1 signifies the absence of pollution [27].

#### 2.3.3. Potential Ecological Risk Index (RI)

The Potential Ecological Risk Index (RI), developed by Hakanson [26], was calculated using the following set of formulas:

(4) $$RI=\sum_{i=1}^{n}E_{r}^{i}$$

(5) $$E_{r}^{i}=T_{r}^{i}\times C_{f}^{i}$$

(6) $$C_{f}^{i}=\frac{C_{n}^{i}}{B_{n}^{i}}$$

where $E_{r}^{i}$, is the potential ecological risk factor for a specific metal, $T_{r}^{i}$ represents the toxic response factor for a specific element, and $C_{f}^{i}$ denotes the Contamination Factor. The $T_{r}^{i}$ for Cd, Co, Cr, Cu, Mn, Ni, Pb, and Zn are 30, 5, 2, 5, 1, 5, 5, and 1, respectively. The $E_{r}^{i}$ value reflects the risk associated with a single element, taking into account its contamination level and toxic response. Based on Hakanson’s classification, an $E_{r}^{i}$ < 40 indicates low risk, values 40 < $E_{r}^{i}$ < 80 signify moderate risk, 80 < $E_{r}^{i}$ < 160 represent a considerable risk, 160 < $E_{r}^{i}$ < 320 denote high risk, and values $E_{r}^{i}$ > 320 indicate a very high ecological risk. The cumulative RI value summarizes the total ecological risk from all analyzed metals. An RI <150 is considered low, 150 < RI < 300 indicates moderate risk, 300 < RI < 600 reflects a considerable ecological threat, and an RI > 600 signifies a very high ecological risk. These thresholds are essential for evaluating pollution severity and prioritizing sites for environmental management or remediation [28,29].

### 2.4. Health Risk Assessment

The potential non-carcinogenic and carcinogenic health risks for both adults and children resulting from exposure to urban soils were assessed using the risk assessment models developed by the United States Environmental Protection Agency [30]. Human exposure was evaluated through two primary pathways: direct ingestion and dermal contact with contaminated soil particles. The average daily doses (ADD) (mg kg^−1^ d^−1^) of each heavy metal via these exposure routes were calculated based on standard equations provided by the USEPA (1989) [31] and are as follows:

(7) $${ADD}_{ing}=\frac{C_{soil}\times IR\times EF\times ED}{BW\times TA}\times CF$$

(8) $${ADD}_{derm}=\frac{C_{soil}\times SA\times AF\times ABS\times EF\times ED}{BW\times TA}\times CF$$

where ADD_ing_ and ADD_derm_ are the daily doses of exposure to metals in urban soils through ingestion and dermal contact, respectively. In this study, ingestion and dermal contact were selected for assessing heavy metal exposure from urban soils, as these routes are widely acknowledged to pose the greatest risk to human health, particularly for children [32]. The parameters used in the models for children and adults were derived from the USEPA Risk Assessment Guidance and are listed in Table 3.

The non-carcinogenic and carcinogenic health risks associated with the analyzed metals were evaluated using the Hazard Quotient (HQ), cumulative Hazard Quotient (HQ_tot_), and carcinogenic risk (CR) indices [33]. Non-carcinogenic risks via ingestion (HQ_ing_) and dermal contact (HQ_derm_) were calculated following the equations:

(9) $${HQ}_{ing}=\frac{{ADD}_{ing}}{{RfD}_{ing}}$$

(10) $${HQ}_{derm}=\frac{{ADD}_{derm}}{{RfD}_{derm}}$$

In this context, RfD_ing_ refers to the oral reference dose (mg kg^−1^ day^−1^), and RfD_derm_ denotes the dermal reference dose (mg kg^−1^ day^−1^). These values represent the maximum acceptable daily intake through ingestion and dermal contact, respectively, that is not expected to cause harmful effects or adverse health outcomes in humans over a lifetime. Hazard Quotient (HQ) values exceeding the safety threshold of 1 indicate the potential risk of negative health effects [30].

The carcinogenic risk (CR) reflects the probability of an individual developing cancer at some point in their lifetime due to long-term exposure to a carcinogenic substance, and it is calculated using the following formula:

(11) $$CR={ADD}_{i}\times {SF}_{i}$$

In this assessment, “i” represents the specific exposure pathways—ingestion and dermal contact, while SFi refers to the slope factor (mg kg^−1^ d^−1^) associated with each route. If the carcinogenic risk (CR) is below 10^−6^, it is considered negligible in terms of potential cancer development due to soil exposure. In contrast, a CR value exceeding 10^−4^ indicates a potentially elevated cancer risk. Values between 10^−6^ and 10^−4^ are typically regarded as an acceptable or tolerable range of risk for public and human health [34]. The RfDs and SFs for each metal are based on the USEPA Regional Screening Level (RSL) tables and are presented in Table 3 [35].

### 2.5. Statistics

Multivariate statistical analyses including the analysis of variance (one-way ANOVA), Principal Component Analysis (PCA), and Hierarchical Cluster Analysis (HCA) were utilized to identify potential sources of the detected metals by reducing the dataset’s dimensionality into a few key components. PCA was performed using Varimax rotation to simplify the interpretation by concentrating variable loadings on fewer factors, retaining only those with eigenvalues greater than 1. The data’s suitability for PCA was confirmed by the Kaiser–Meyer–Olkin (KMO) measure of sampling adequacy, which was 0.752, and Bartlett’s Test of Sphericity, which yielded a chi-square value of 1134.5 (d_f_ = 28, *p* < 0.001), indicating sufficient correlations among variables for the PCA. HCA was carried out using Ward’s linkage method, with the squared Euclidean distance as the metric for measuring similarity. Data were standardized to z-scores before clustering, and the results were visualized in a dendrogram. Moreover, all statistical analyses were performed using SPSS software (version 29.0, IBM Corp., Armonk, NY, USA). Additionally, the spatial distribution of contamination and health risk indices were illustrated through QGIS. The method used for the spatial distribution of parameters is Ordinary Kriging, which is a geostatistical interpolation method used to estimate the value of a variable at an unobserved location based on values at nearby known locations. It assumes that the mean of the variable is constant but unknown in the area being interpolated. This method relies on a variogram, which describes the spatial correlation between data points, to determine the weights used in the estimation.

## 3. Results and Discussion

### 3.1. Physicochemical Properties of Urban Soils

Table 1 provides the boundary values of the physicochemical properties of the soil samples. Thus, the soils have a minimum pH value of 7.5, which coincides with Sager’s research wherein the high pH values of the urban soil samples and their alkaline reaction generally are explained [36]. In the current investigation, 53.9% of soil samples have values between 8 and 8.5, while 19.2% are above 8.5. The electrical conductivity (EC) values show significant variability as they range from 95.6 to 1431.5 μS/cm; however, the EC values do not indicate saline or sodic soils [37]. About 11.5% of the soil samples show EC values greater than 1000 μS/cm. Nonetheless, a high pH value coupled with a subsequent rise in conductivity is a reason to be concerned, especially if these soils are continuously fertilized to improve the aesthetics of the city’s green spaces [38]. On the other hand, the variety of construction materials, such as the cement in pavements or flowerbeds possibly added to soils filling the small parks and green spaces in the study area, provoke both an increase in the soil pH as well as in soil salinity [39].

In terms of soil texture, 46.2% are Sandy Loam, whereas 32.7% are characterized as Loamy soils. In the previous study’s [40] survey, they established that urban soils have a high percentage of loam and are mainly sand, resulting in soils that can easily lose their moisture, which requires frequent watering care and also the addition of fertilizer because of nutrient leaching.

### 3.2. Concentrations of Heavy Metal Contents in Urban Soils

Descriptive statistics of heavy metal contents in the investigated urban soils are presented in Table 1. There were significant variations among the heavy metal contents with ranges of 0.5–1.8 mg kg^−1^ for Cd, 6.6–33.5 mg kg^−1^ for Co, 19.5–112.4 mg kg^−1^ for Cr, 17.5–93.6 mg kg^−1^ for Cu, 183.6–985.6 mg kg^−1^ for Mn, 17.4–71.1 mg kg^−1^ for Ni, 9.4–61.7 mg kg^−1^ for Pb, and 29–302.6 mg kg^−1^ for Zn. The mean concentrations decreased in the order Mn > Zn > Cr > Cu > Ni > Pb > Co > Cd, with the mean contents of Mn and Zn being significantly higher than the other metals studied, while Cd exhibited the lowest concentrations. Compared with the background values in Upper Continental Crust [41], the mean concentrations of Co, Cr, Mn, and Ni were lower than the background values; however, 26, 16, and 26% of the sampled soils are exceeding the crust values for Co, Cr, and Ni, respectively. On the other hand, the mean contents of Cd, Cu, Pb, and Zn were approximately 10.2, 1.7, 1.9, and 1.8 times higher than their background values, respectively, possibly showing that the investigated soil samples were probably influenced by human activities [42]. Coefficients of variation (CV) can be used to evaluate the average level of variability in samples. A CV less than 15% suggests low variability, a CV between 15% and 35% indicates moderate variability, and a CV greater than 35% reflects high variability. The CVs of all studied metals were above 35% indicating high variability, which may suggest a notable influence from human activities [42,43].

The urban soils of Thessaloniki exhibit a distinct heavy metal profile when compared with other urban environments both within Greece and globally. Overall, the levels of potentially toxic elements reflect a moderate contamination pattern characteristic of urban areas influenced by vehicular traffic, industrial activities, and historical urbanization. Compared to other Greek cities, Thessaloniki shows comparable or elevated concentrations for several metals. For instance, the heavy metal load in Thessaloniki is similar to that observed in Volos [44] and Drama [45], with comparable Co, Cr, Cu, Mn, and Pb levels but remains lower than the profile reported for Athens [46] and Lamia [47], where notably elevated Cr and Ni concentrations were recorded as linked to geogenic influences. When compared with urban agglomerations abroad, Thessaloniki’s soil metal content is generally lower than that of heavily industrialized or traffic-impacted urban centers such as Napoli [48] and Bristol [49]. These cities report significantly elevated Pb and Zn concentrations, indicative of persistent pollution from past leaded gasoline usage and dense industrial activity. For instance, Napoli recorded Pb and Zn levels of 141 mg/kg and 272.6 mg/kg, respectively, far exceeding those in Thessaloniki. In contrast, Lisbon [50] showed lower concentrations for almost all metals (except Pb).

Of particular importance are the elevated Cd contents observed in the studied urban soils. The median Cd levels (0.9 mg/kg) were among the highest recorded in Greece, surpassing values reported by [46] for Athens (0.3 mg/kg), by [47] for urban soils from Lamia city (0.22 mg/kg), and also in soil samples from Volos (0.27 mg/kg) and Drama (0.28 mg/kg) by [44,45], respectively. Yet, they were only lower than the concentrations reported in Bristol, UK [49]. Although the Cd levels recorded align with the typical abundances reported for urban environments [51], this does not negate the environmental concern, as Cd is a non-essential, highly toxic element with strong ecological and human health implications, particularly when bioavailable. This underscores that while Thessaloniki does not rank among the most contaminated cities, it nonetheless exhibits pollutant levels that warrant continuous monitoring, especially in light of urban expansion and ongoing anthropogenic inputs.

### 3.3. Seasonal Variation in Heavy Metals in Urban Soils

**Figure 2:**
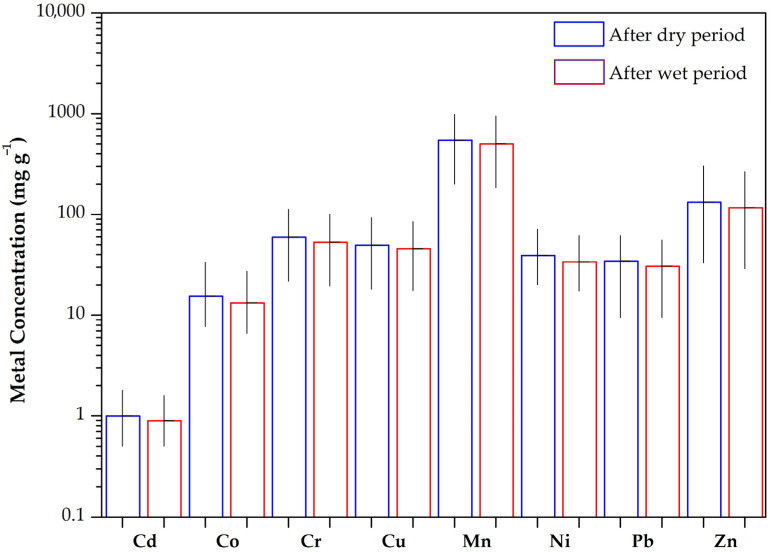
Variations in heavy metal contents among sampling periods for urban soils from Thessaloniki, Greece.

The seasonality of heavy metal contents in urban soils reveals significant temporal variations, often linked to changes in environmental conditions and human activities. The heavy metal concentrations at different sampling periods are given in Table 1 and illustrated in Figure 2.

**Table 1:** Summary statistics of physicochemical characteristics and heavy metal concentrations in urban soils from the city of Thessaloniki during the two distinct sampling periods (summer—S and winter—W). Background values and the literature data on published heavy metal median concentrations (in mg kg^−1^) in urban soils from various cities around the world are also included.

	pH	EC	Cd		Co		Cr		Cu		Mn		Ni		Pb		Zn	
Sampling Period			S	W	S	W	S	W	S	W	S	W	S	W	S	W	S	W
Min	7.5	95.6	0.5	0.5	7.7	6.6	21.7	19.5	18.1	17.5	199.1	183.6	20	17.4	9.4	9.5	33.2	29
Max	9.0	1431.5	1.8	1.6	33.5	27.4	112.4	100.7	93.6	84.9	985.6	949.7	71.1	62	61.7	55.6	302.6	266.3
Mean	8.2	481.6	1.0	0.9	15.5	13.3	59.7	53.1	49.7	45.6	545.9	501.3	39.3	33.8	34.4	30.7	132.3	116.5
Total mean content (n = 104)	8.2	481.6	0.9		14.4		56.4		47.7		523.6		36.6		32.6		124.4	
Median	8.2	349.7	0.9		13.3		51.3		43.5		482.7		33.8		31.8		98.4	
SD	0.4	361.6	0.4	0.3	6.6	5.5	28.8	25.5	24.0	21.5	262.0	240.8	15.7	13.5	16.5	14.6	90.1	79.2
CV			39.7	37.4	42.5	41.8	48.3	48.0	48.3	47.1	48.0	48.0	39.8	40.1	48.0	47.7	68.1	68.0
Total CV			39.3		42.9		48.3		47.7		48.0		40.6		48.1		68.2	
Earth’s crust ^a^			0.09		17.3		92		28		1000		47		17		67	
Earth’s soils ^b^			0.5		8		200		20		850		40		10		50	
Urban soils abundances ^b^			0.9		14.1		80		39		729		33		54.5		158	
EU limits			3						140						300		300	
Athens, Greece ^1^			0.3		16		141		39		554		102		45		98	
Lamia, Greece ^2^			0.22		37.8		258		59.4		1081		409		15.1		77	
Larissa, Greece ^3^			0.09						47.00						43.24		84.60	
Volos, Greece ^4^			0.27		15.7		71		42.6		607		68.9		29.5		99.0	
Drama, Greece ^5^			0.28		18.0		63.9		19.5		578		41.3		27.6		59.7	
Shanghai, China ^6^			0.61						55.8				89.9		42.4		77.4	
Lisbon, Portugal ^7^					6.8		16		29		218		20		62		88	
Bristol, UK ^8^			1.1				23.1		60.1				21		210.1		272.6	
Napoli, Italy ^9^			0.37		6.3		11.2		74		635		8.9		141		158	

^a^ [41], ^b^ [51], ^1^ [46], ^2^ [47], ^3^ [13], ^4^ [44], ^5^ [45], ^6^ [52], ^7^ [50], ^8^ [49], ^9^ [48].

As shown, heavy metals revealed a clear pattern of slightly increased concentrations during the summer. Specifically, zinc mean concentrations were marginally higher after the dry period (132 mg kg^−1^) compared to after the wet sampling period (116.5 mg kg^−1^), while cadmium was found to have similar concentrations in both sampling periods (1.0 and 0.9 mg kg^−1^, respectively). The mean concentrations of Pb and Cu, which are associated with traffic [52], were also slightly higher during the summer, with concentrations ranging from 9.4 to 61.7 mg kg^−1^ and from 18.1 to 93.6 mg kg^−1^, respectively, as compared to the winter sampling period (9.5–55.6 mg kg^−1^ and 17.5–84.9 mg kg^−1^, respectively). Although noticeable differences in heavy metal concentrations were observed, these differences were statistically insignificant (one-way ANOVA, *p* >0.05), indicating that the observed variations may not reflect a definite seasonal effect and interpretations should be made with caution due to the lack of statistical significance. However, this seasonal rise is likely driven by factors such as enhanced atmospheric deposition from intensified human activities, reduced soil moisture leading to limited metal mobility, and increased resuspension of contaminated dust due to dry conditions [16]. Additionally, higher temperatures can accelerate the weathering of building materials and road surfaces, contributing further to the accumulation of metals like Pb, Zn, and Cu in urban soils [53,54]. Nonetheless, these findings highlight the importance of considering seasonal influences when assessing urban soil contamination and formulating effective environmental management strategies, even if the seasonal effects observed here remain inconclusive.

### 3.4. Pollution Assessment

#### 3.4.1. Geoaccumulation Index (I_geo_)

**Figure 3:**
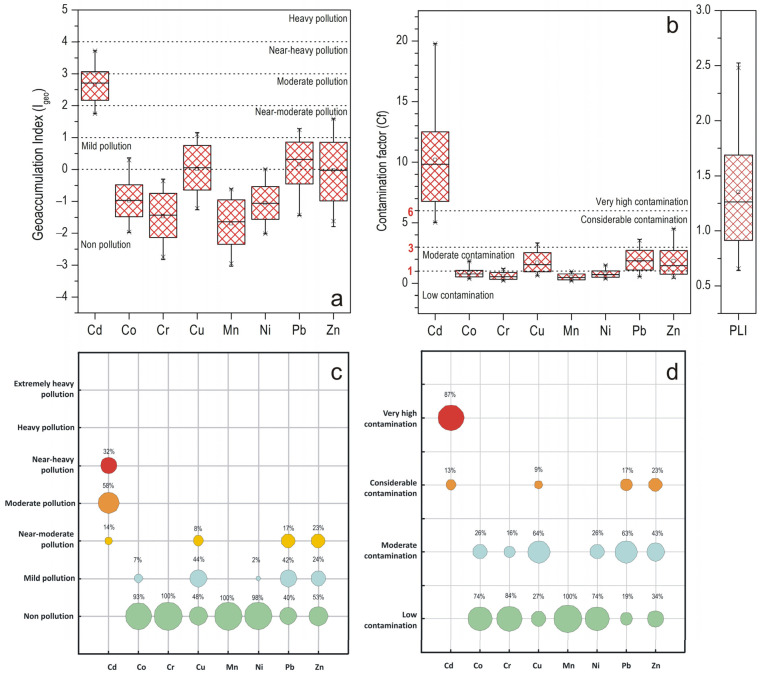
Contamination assessment results. (**a**) Boxplot of Geoaccumulation Index (I_geo_) of heavy metals. (**b**) Boxplot of Contamination Factor (C_f_) and Pollution Load Index (PLI) of heavy metals. (**c**) Proportion chart of I_geo_ of heavy metals. (**d**) Proportion chart of Cf of heavy metals. Different colors indicate different levels of heavy metal pollution.

The classification of urban soils contamination based on the computed I_geo_ values reveals distinct patterns among the investigated heavy metals as presented in Figure 3. As seen, mean I_geo_ values exhibit a descending order as follows: Cd >> Pb > Zn > Cu > Co> Ni >Cr > Mn. Overall, the I_geo_ assessment indicated Cd as the most prominent pollutant in the studied urban soils, with 30.8% of the samples categorized as “near-heavy polluted” and 55.8% as “moderate polluted,” indicating significant anthropogenic enrichment. On the other hand, the I_geo_ values of Cu, Pb, and Zn reflect a moderate degree of pollution, indicating notable anthropogenic influence. Specifically, 44.2% of samples were classified as “mildly polluted” with respect to Cu, while an additional 7.7% fell under the “near-moderate pollution” category, suggesting a moderate but widespread presence of Cu in urban soils. Regarding Pb, a historically persistent urban contaminant, 42.3% of sampled soils were classified as “mildly polluted” and 17.3% as “near-moderately polluted”, while for Zn, 24.0% and 23.1% of the samples were identified as “mildly” and “near-moderately polluted,” respectively. In contrast, Co, Cr, Mn, and Ni appear to be primarily of geogenic origin with negligible pollution influence. Co and Cr showed minimal contamination; 93.3% and 100% of the samples, respectively, were classified as “non-polluted,” with only a minor fraction of sampled soils (6.7%) falling into the “mild polluted” category with respect to Co. Urban soils also displayed low contamination levels with respect to Mn and Ni, with 100% and 98.1% of the sampled soils, respectively, falling into the “non-polluted” class.

#### 3.4.2. Contamination Factor (Cf)

The Cf values for heavy metals in the analyzed urban soils were determined, with the results presented in Figure 3. The mean Cf values followed a descending trend Cd >> Pb > Cu > Zn > Co > Ni > Cr > Mn, with Cd showing again the most alarming contamination profile, with almost all samples (87%) exhibiting values greater than six classifying them as highly contaminated, underscoring its dominant anthropogenic origin. In contrast, Cu, Pb, and Zn demonstrated a broader distribution with 65%, 58%, and 42%, respectively, of the samples characterized as moderately contaminated (1 < CF < 3), reflecting consistent anthropogenic input. However, a limited number of sampled soils (in 7 samples for Cu, while Pb and Zn each presented 13 samples) were characterized as considerably contaminated, highlighting localized hotspots of contamination. Regarding Co and Mn, urban soils were categorized as moderately contaminated consistent with a moderate anthropogenic influence, while Cr and Ni presented the lowest contamination signatures, with the majority of the sampled soils classified as uncontaminated confirming their predominantly lithogenic origin.

To evaluate the cumulative contamination from multiple heavy metals, the Tomlinson Pollution Load Index (PLI) was employed. As shown in Figure 3, 70% of the sampling sites recorded PLI values greater than 1, ranging between 1.07 and 2.52, signifying a moderately polluted environment.

**Figure 4:**
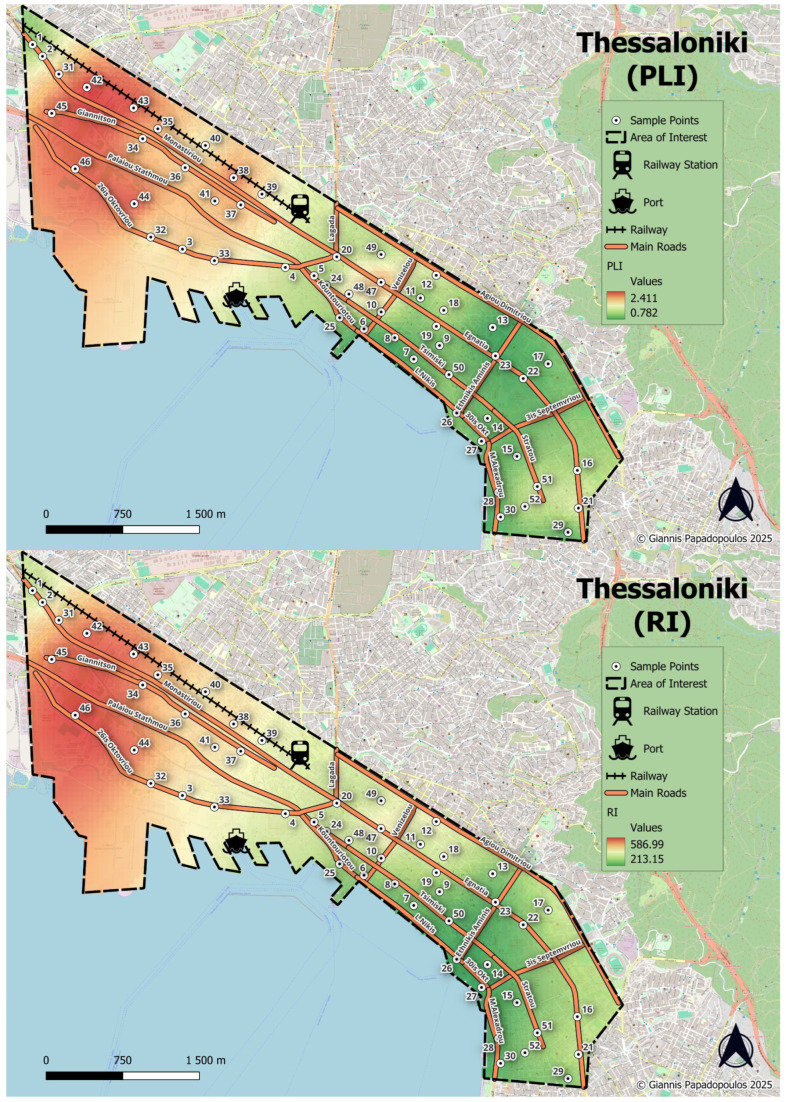
Spatial distribution of Pollution Load Index—PLI (**up**) and Potential Ecological Risk Index—RI (**down**).

The spatial distribution of PLI, given in Figure 4, reveals a distinct west-to-east gradient, with higher PLI values in the northwestern and central-western parts of the city, particularly observed in areas adjacent to the port, major roadways, and the railway network indicating the significant influence of industrial activities and transportation. Moreover, high to moderate PLI values were observed in the historic urban center, especially along major arterial roads reflecting the impact of dense traffic and urban activity. On the contrary, the southeastern suburbs of the city, consisting of more residential and green areas, exhibit notably lower PLI values.

#### 3.4.3. Potential Ecological Risk Index (RI)

**Figure 5:**
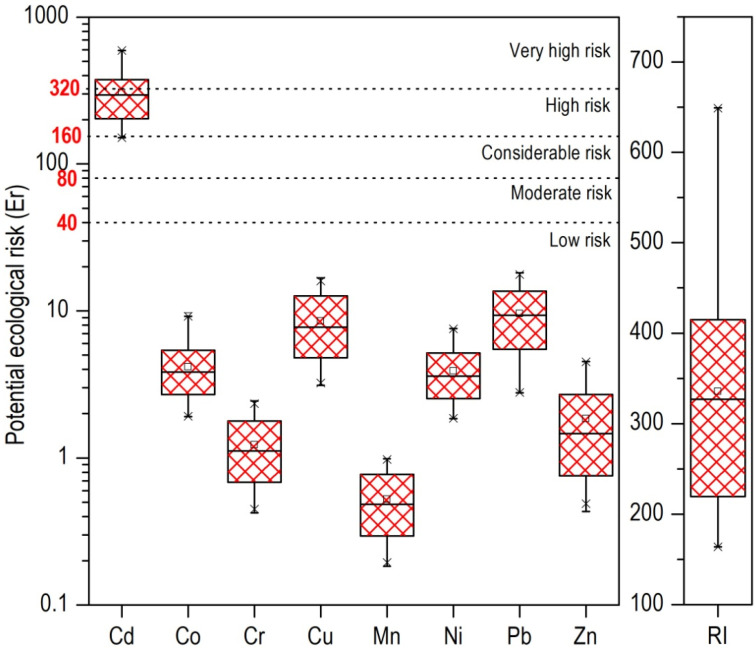
Potential ecological risk (Er) and cumulative ecological risk index (RI) for the studied heavy metals in urban soils from the city of Thessaloniki.

To gain a more comprehensive understanding of the contamination levels and their associated ecological threats, the potential ecological risk was assessed, with the results presented in Figure 5. Cd was revealed as the primary contributor to ecological risk in the study area. As shown, all soil samples exhibited Er values for Co, Cr, Cu, Mn, Ni, Pb, and Zn below the threshold of Er < 40, indicating low risk. In contrast, Cd showed markedly elevated Er values, ranging from 151 to 593. Similarly to our study, elevated Er values for Cd were reported by Siddig et al. [55] in anthropogenically affected soils of Sudan. Almost all of the sampled soils (except five samples) exhibited Er values well above 160, signifying a high to extreme ecological risk. This highlights a site-wide concern for Cd contamination, as it was the only one of the studied heavy metals to pose significant potential ecological risks [55].

The overall Potential Ecological Risk Index (RI) for the studied heavy metals further corroborates this finding. Values ranging from 163.9 to 649.2, with an average of 359.2, were recorded, while Cd was the main contributor to RI. Half of the sampled soils exhibited considerable ecological risk (RI = 300–600), while five samples computed RI values greater than 600, indicating a high level of ecological risk. Spatially, the distribution of the RI (Figure 4) mirrors that of the PLI, revealing also a pronounced west-to-east gradient.

The sampling sites in the northwestern sector of the city exhibited higher RI values with those surrounding the bus station and sites close to the railway presenting significant hotspots due to intense industrial, transport, and port-related activities. Additionally, parks located near the city port have also been identified as critical zones, exhibiting elevated RI values suggesting potential ecological threats. These results underscore the need for targeted mitigation strategies, particularly addressing Cd pollution in urban soils. Comparing this with other related studies, Shen et al. [56] assessed the potential ecological risk of heavy metals in urban soils in Shanghai, finding RI values between 35.28 and 3974.95, signifying high ecological risks due to elevated concentrations of Hg and Cd. In the urban soils of Kaifeng (Henan Province), a very high ecological risk was reported by Cd with the mean value of the RI to be 344.58 [57].

### 3.5. Source Identification

To assess the potential sources of the analyzed elements, Principal Component Analysis (PCA) was conducted on the complete dataset of urban soil samples collected from both sampling periods. The PCA outcomes, including component loadings, total eigenvalues, and the explained variance percentages for the full dataset, are shown in Table 2. Two main components were obtained accounting for 69.3% of the total variance, and the eigenvalues of the extracted factors were above one. Component 1 exhibited strong positive loadings for Ni (0.889), Co (0.877), and Pb (0.826), suggesting mixed natural and anthropogenic inputs. Cadmium, although moderately loaded on Component 1 (0.644), showed relatively weak associations elsewhere. The co-loading of Pb and Cd along with the elevated contamination indices recorded (Figure 3) implicate anthropogenic sources, as these elements are frequently linked to fossil fuel combustion and vehicular wear (e.g., tires, brakes, and lubricants), all of which are typical contributors to urban soil contamination [58,59,60]. On the other hand, despite their prominent roles in Component 1, Co and Ni demonstrated consistently low I_geo_ and Cf values (Figure 3) indicating minimal anthropogenic input. Thus, the strong association between Co and Ni likely reflects a shared geogenic origin, potentially linked to the regional geology, as reported in previous studies [59,61]. Moreover, these elements are also commonly incorporated into iron and manganese oxides, further contributing to their co-mobilization under stable redox conditions.

Component 2 displays high loadings for Mn (0.958), Cu (0.958), and, to a lesser extent, Zn (0.494). Mn was a dominant component in this factor and demonstrated consistently low I_geo_ and Cf values (Figure 3). Thus, its strong association with Cu (co-loading) may reflect geochemical processes such as redox-controlled mobility or interactions with Fe/Mn oxides that are common in soil environments rather than direct anthropogenic inputs. This behavior is consistent with Mn’s natural affinity for oxide phases and its geochemical stability in oxidizing urban soil environments. Zinc’s moderate cross-loading may reflect its widespread presence in urban environments [62]. Overall, the PCA results underscore that it is not reserved only to group metals by common sources but also to highlight the geochemical coherence of elements like Co, Ni, and Mn, whose low pollution levels suggest that their presence in urban soils is primarily lithogenic rather than anthropogenic.

**Figure 6:**
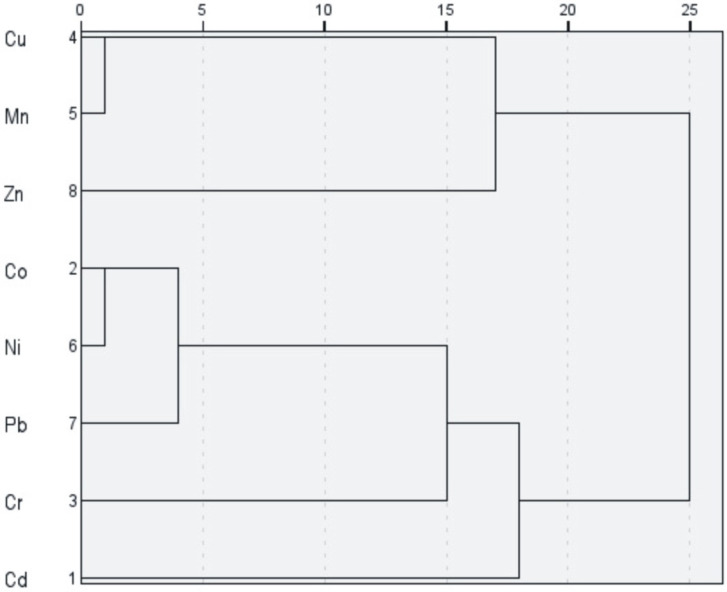
Hierarchical Cluster Analysis (HCA) classification of heavy metals in groups based on z-score similarities.

The Hierarchical Cluster Analysis (Figure 6) revealed a clustering pattern that is broadly consistent with the associations identified in the PCA results. Three distinct clusters were observed: the first containing Cu-Mn-Zn, the second Co-Ni-Pb, while Cr and Cd form a distinct branch, separated at higher linkage distances in line with PCA findings. The isolated positioning of Cd in both PCA and HCA likely reflects its unique anthropogenic input profile, particularly its association with potentially harmful contamination as indicated by its elevated I_geo_ and Cf values. Cr, which displayed moderate enrichment in some samples, could also be influenced by both geogenic sources and localized anthropogenic inputs.

### 3.6. Health Risk Assessment

**Figure 7:**
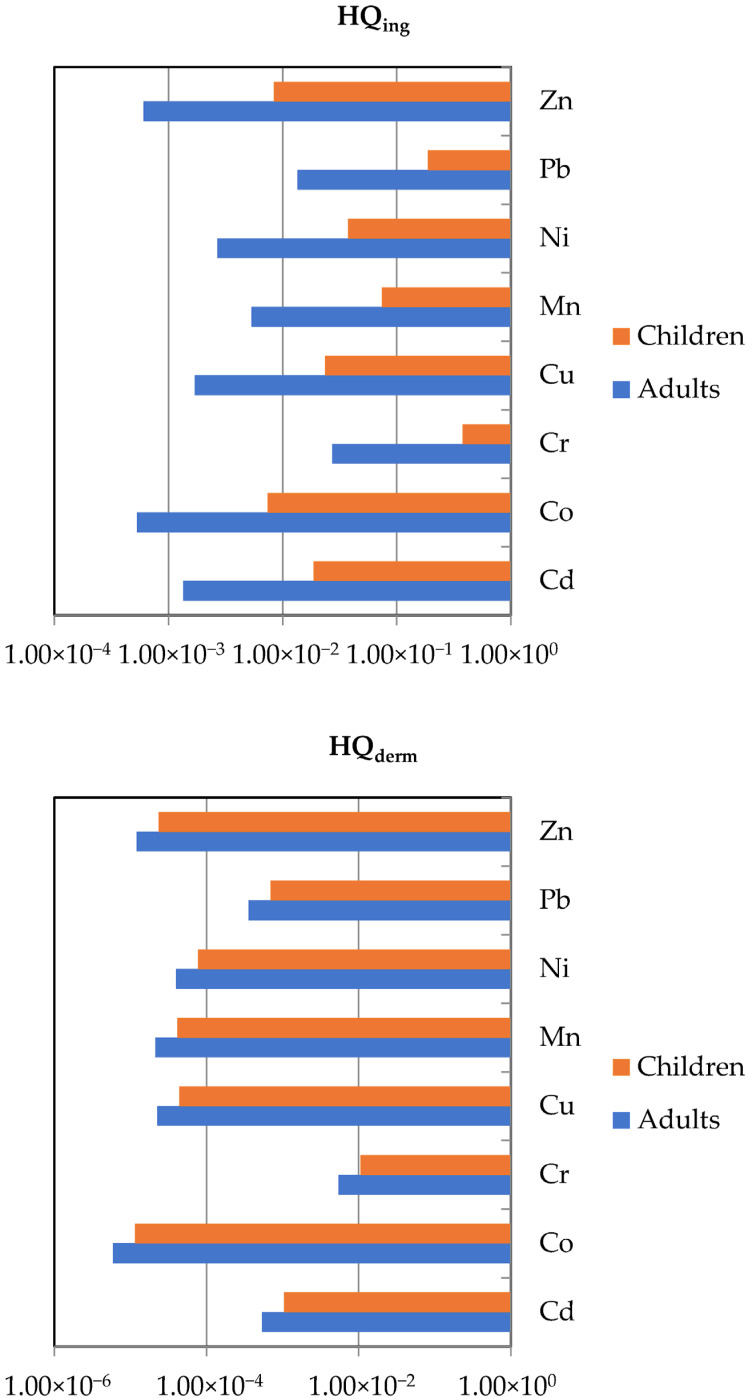
Average non-carcinogenic health risks (HQs) of exposure via ingestion (**up**) and dermal contact (**down**) to heavy metals in urban soils from the city of Thessaloniki.

The health risks associated with heavy metal exposure in the studied urban soils were evaluated for each soil sample, taking into account adults and children. The calculated Hazard Quotients (HQs) and cancer risks (CRs) are summarized in Table 3, while the average HQs are displayed in Figure 7. The results revealed clear age-related differences in exposure and health vulnerability, with children demonstrating consistently higher HQ values than adults across all studied heavy metals in line with previous studies [16,63]. Moreover, the computed HQs for both adults and children followed the sequence: ingestion > dermal contact indicating ingestion to be the dominant exposure route for all the heavy metals analyzed.

Regarding exposure through unintentional ingestion, the average HQs for both adults and children followed a descending order of Cr > Pb > Mn > Ni > Cu > Cd > Co > Zn. Among individual metals, Cr and Pb posed the greatest non-carcinogenic risk to children, with maximum HQ values of 4.79 × 10^−1^ and 2.25 × 10^−1^, respectively, both approaching the risk threshold of unity. Similarly, the cumulative non-carcinogenic risk ranged from 1.96 × 10^−1^ to 8.73 × 10^−1^ (mean: 4.94 × 10^−1^), suggesting a moderate potential for adverse health effects, especially in locations where contamination is more pronounced. For adults, HQ values remained well below the threshold of concern and ranged from 6.04 × 10^−4^ (Zn) to 2.72 × 10^−2^ (Cr), indicating negligible non-carcinogenic risk through soil ingestion. The values of cumulative HQ were considerably lower, ranging from 2.10 × 10^−2^ to 9.35 × 10^−2^ (mean: 5.29 × 10^−2^), indicating minimal risk from incidental soil ingestion. Nonetheless, Cr and Pb again represented the dominant contributors, aligning with their elevated toxicity and environmental persistence, despite Cd being identified as the main contributor to contamination based on contamination indices.

Regarding dermal exposure to heavy metals, the average HQs revealed a descending order of Cr > Cd > Pb > Ni > Cu > Mn > Zn > Co for both adults and children. Chromium (Cr) exhibited the highest dermal HQs for both adults (5.44 × 10^−3^) and children (3.56 × 10^−2^), followed by cadmium (Cd) and lead (Pb). In children, Cd (3.52 × 10^−3^) and Pb (2.35 × 10^−3^) also contributed notably to dermal risk. Although all average HQs were below the threshold value of one, indicating no significant non-carcinogenic risk through dermal contact, the relatively elevated values of Cr, Cd, and Pb—particularly in children—suggest that these elements warrant closer attention in future risk management and soil remediation strategies.

For carcinogenic risk, the CRs obtained for Cd and Pb were within the acceptable safety limits (10^−6^ to 10^−4^). However, CR values for chromium ranged from 4.28 × 10^−4^ to 2.22 × 10^−3^, with a mean of 1.77 × 10^−3^, all of which exceed the acceptable risk limits, indicating a high potential carcinogenic risk associated with long-term Cr exposure through soil ingestion, warranting urgent attention and possible remediation.

**Figure 8:**
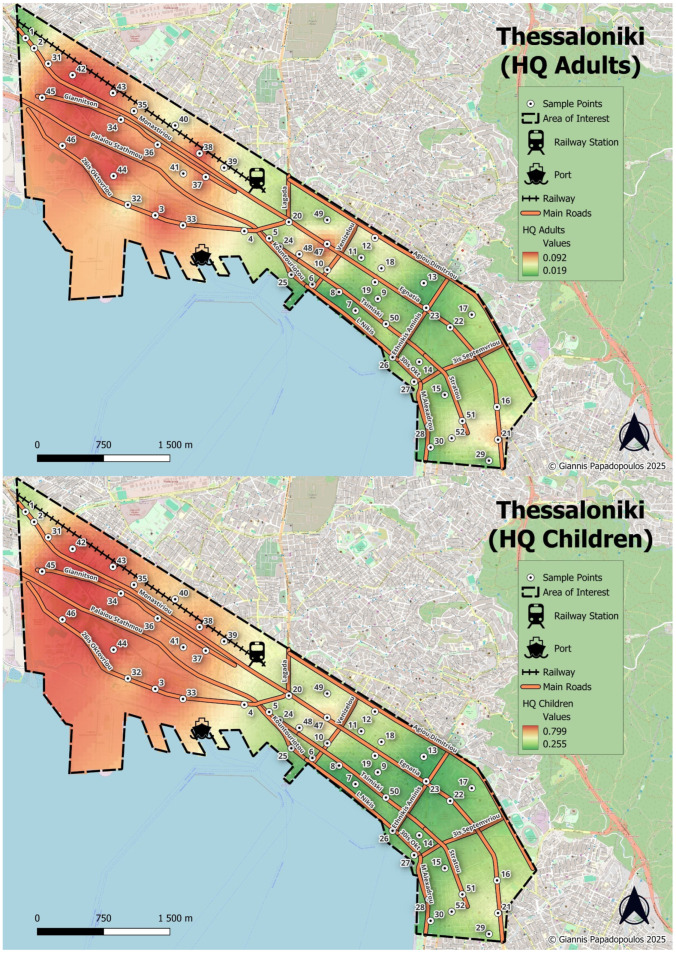
Spatial distribution of cumulative HQ through ingestion for adults (**up**) and children (**down**).

The spatial distribution of the non-carcinogenic cumulative HQs through ingestion (as the main exposure pathway) for both adults and children is presented in Figure 8. Though the HQ values remain below the threshold of unity, the spatial pattern reveals notable areas of concern. Specifically, elevated HQs, reaching up to 0.873, are concentrated in the northwestern areas of the city coinciding with zones of elevated PLI and RI values (Figure 8), highlighting a consistent accumulation of heavy metals and the associated ecological pressures. Despite HQ values suggesting potentially low health risks, the co-occurrence of high pollution loads and ecological risks suggest cumulative environmental stress that could affect vulnerable populations, particularly children, over prolonged periods. Moreover, the elevated carcinogenic risk for chromium necessitates risk management interventions, particularly in areas with a high potential of exposure to children such as playgrounds, schools, or residential backyards. 

Our findings are consistent with previous research conducted by Gopal et al. in Southern India, where Cr contributed most prominently to the total hazard index (HI), with HQ values for children reaching 0.561 through ingestion, while HQ through dermal exposure was up to 0.068, exceeding values reported for other elements like Pb and As [64]. Chromium’s elevated risk levels in urban soils have also been reported in Pan et al. [65] where in several Chinese cities Cr posed potential cancer risks to both adults and children. Similarly, Soltani-Gerdefaramarzi et al. [59] found that Cr posed a potential cancer risk, particularly for children, with cancer risks exceeding the acceptable threshold of 1 × 10^−4^. This is consistent with the classification of Cr^6+^ as a Group 1 carcinogen by the IARC, with exposure routes including ingestion and dermal absorption [66]. Moreover, prenatal and early-life exposure to Cr has been associated with developmental toxicity and congenital abnormalities [67]. These converging findings underline the widespread concern over chromium contamination in urban environments and emphasize the necessity of targeted monitoring and mitigation strategies to address long-term public health implications, particularly for vulnerable populations like children.

## 4. Conclusions

The findings of this study reveal that urban and peri-urban soils in Thessaloniki are subject to substantial heavy metal contamination, with averages exhibiting a descending order of Mn > Zn > Cr > Cu > Ni > Pb > Co > Cd, with Mn and Zn being significantly higher than the other metals studied. Notably, cadmium (Cd) levels are of particular concern, as several locations exhibited Cd concentrations exceeding both crustal background values and patterns reported for other major urban agglomerations, indicating that though Thessaloniki is not classified among the most heavily polluted cities, it still displays contamination levels that justify ongoing surveillance, particularly considering urban growth and persistent human activities. While slight increases in heavy metal contents were observed following the dry summer period, these seasonal differences were not statistically significant and should therefore be interpreted with caution. Nonetheless, environmental factors such as reduced soil moisture and increased atmospheric deposition during the dry season may play a role and warrant further investigation. Pollution indices, including I_geo_, Cf, and RI, confirm that while most sites are classified as uncontaminated to moderately contaminated, certain hotspots present considerable to very high contamination and ecological risk with Cd identified as the most prominent pollutant followed by Cu, Pb, and Zn. Multivariate statistical analyses further demonstrate that heavy metal contamination in Thessaloniki’s soils is driven by both anthropogenic sources—such as traffic and industrial emissions—and natural geogenic factors, a pattern also observed in comparable European cities. Health risk assessments show that non-carcinogenic risks for adults and children generally remain below critical thresholds; however, in areas with the highest contamination, children are at increased risk due to greater exposure through ingestion and dermal contact. Collectively, these results underscore the urgent need for ongoing monitoring and targeted remediation in identified hotspots to mitigate ecological and health risks. This study provides a critical baseline for future research and policy development aimed at managing urban soil pollution and protecting public health in Thessaloniki and similar urban environments.

## Figures and Tables

**Table 2:** Main group components derived by Principal Component Analysis (PCA). Values in bold represent strong correlation, while underlined represent moderate correlation.

Elements	Component	
	1	2
Cd	0.644	−0.117
Co	**0.877**	0.334
Cr	0.466	0.322
Cu	0.143	**0.958**
Mn	0.147	**0.958**
Ni	**0.889**	0.359
Pb	**0.826**	0.371
Zn	0.228	0.494
Eigenvalue	4.17	1.373
% variance explained	52.126	17.161
Cumulative % variance	52.126	69.287

**Table 3:** Non-carcinogenic and carcinogenic risks of each element and exposure pathway for urban soils from the city of Thessaloniki, Greece. Underlined the values which are close to the risk threshold of unity.

Elements	RfD_ing_	RfD_derm_	SF_o_			HQ_ing_	HQ_derm_		HQ_ing_	HQ_derm_			CR
Cd	1.00 × 10^−3^	1.00 × 10^−5^	6.10 × 10^0^	Min	**Adults**	6.85 × 10^−4^	2.73 × 10^−4^	**Children**	6.39 × 10^−3^	1.79 × 10^−3^	Cd_carc_	Min	1.43 × 10^−6^
				Max		2.44 × 10^−3^	9.73 × 10^−4^		2.28 × 10^−2^	6.37 × 10^−3^		Max	5.1 × 10^−6^
				Mean		1.35 × 10^−3^	5.37 × 10^−4^		1.87 × 10^−2^	3.52 × 10^−3^		Mean	2.82 × 10^−6^
Co	2.00 × 10^−2^	1.60 × 10^−2^		Min		2.63 × 10^−4^	3.53 × 10^−6^		2.45 × 10^−3^	2.31 × 10^−5^			
				Max		1.15 × 10^−3^	9.67 × 10^−6^		1.07 × 10^−2^	6.33 × 10^−5^			
				Mean		5.32 × 10^−4^	5.91 × 10^−6^		7.37 × 10^−3^	3.87 × 10^−5^			
Cr	3.00 × 10^−3^	6.00 × 10^−5^	4.20 × 10^1^	Min		9.92 × 10^−3^	1.98 × 10^−3^		9.26 × 10^−2^	1.30 × 10^−2^	Cr_carc_	Min	4.28 × 10^−4^
				Max		5.13 × 10^−2^	1.02 × 10^−2^		4.79 × 10^−1^	6.70 × 10^−2^		Max	2.22 × 10^−3^
				Mean		2.72 × 10^−2^	5.44 × 10^−3^		3.78 × 10^−1^	3.56 × 10^−2^		Mean	1.18 × 10^−3^
Cu	4.00 × 10−2	1.20 × 10^−2^		Min		6.20 × 10^−4^	8.24 × 10^−6^		5.79 × 10^−3^	5.40 × 10^−5^			
				Max		3.21 × 10^−3^	4.27 × 10^−5^		2.99 × 10^−2^	2.79 × 10^−4^			
				Mean		1.70 × 10^−3^	2.27 × 10^−5^		2.36 × 10^−2^	1.48 × 10^−4^			
Mn	1.40 × 10^−1^			Min		1.95 × 10^−3^	7.77 × 10^−6^		1.82 × 10^−2^	5.09 × 10^−5^			
				Max		9.64 × 10^−3^	3.85 × 10^−5^		9.00 × 10^−2^	2.52 × 10^−4^			
				Mean		5.34 × 10^−3^	2.13 × 10^−5^		7.41 × 10^−2^	1.40 × 10^−4^			
Ni	2.00 × 10^−2^	5.40 × 10^−3^		Min		1.37 × 10^−3^	2.02 × 10^−5^		1.28 × 10^−2^	1.32 × 10^−4^			
				Max		4.87 × 10^−3^	7.20 × 10^−5^		4.55 × 10^−2^	4.72 × 10^−4^			
				Mean		2.69 × 10^−3^	3.98 × 10^−5^		3.74 × 10^−2^	2.61 × 10^−4^			
Pb	3.50 × 10^−3^	5.25 × 10^−4^	8.50 × 10^−3^	Min		3.68 × 10^−3^	9.80 × 10^−5^		3.44 × 10^−2^	6.42 × 10^−4^	Pb_carc_	Min	3.76 × 10^−8^
				Max		2.42 × 10^−2^	6.43 × 10^−4^		2.25 × 10^−1^	4.21 × 10^−3^		Max	2.46 × 10^−7^
				Mean		1.35 × 10^−2^	3.59 × 10^−4^		1.87 × 10^−1^	2.35 × 10^−3^		Mean	1.37 × 10^−7^
Zn	3.00 × 10^−1^	6.00 × 10^−2^		Min		1.52 × 10^−4^	3.02 × 10^−6^		1.41 × 10^−3^	1.98 × 10^−5^			
				Max		1.38 × 10^−3^	2.76 × 10^−5^		1.29 × 10^−2^	1.81 × 10^−4^			
				Mean		6.04 × 10^−4^	1.21 × 10^−5^		8.38 × 10^−3^	7.89 × 10^−5^			
HQ_tot_				Min		2.10 × 10^−2^	2.55 × 10^−3^		1.96 × 10^−1^	1.67 × 10^−2^			
				Max		9.35 × 10^−2^	1.15 × 10^−2^		8.73 × 10^−1^	7.55 × 10^−2^			
				Mean		5.29 × 10^−2^	6.43 × 10^−3^		4.94 × 10^−1^	4.21 × 10^−2^			

## Data Availability

Data that support the findings of this study are available from the corresponding author upon reasonable request.

## References

[1] Awewomom J., Dzeble F., Takyi Y.D., Ashie W.B., Ettey E.N.Y.O., Afua P.E., Sackey L.N.A., Opoku F., Akoto O. (2024). Addressing global environmental pollution using environmental control techniques: A focus on environmental policy and preventive environmental management. Discov. Environ..

[2] Xu X., Zhong Y., Cai S., Lei L., Peng J. (2025). Does Air Pollution Aggravate Health Problems in Low-Income Countries? Verification from Countries Along the Belt and Road. Sustainability.

[3] Meravi N., Singh P.K., Prajapati S.K. (2021). Seasonal variation of dust deposition on plant leaves and its impact on various photochemical yields of plants. Environ. Chall..

[4] Arshad K., Hussain N., Ashraf M.H., Saleem M.Z. (2024). Air pollution and climate change as grand challenges to sustainability. Sci. Total Environ..

[5] Dawam M., Gobara M., Oraby H., Zorainy M.Y., Nabil I.M. (2025). Advances in Membrane Technologies for Heavy Metal Removal from Polluted Water: A Comprehensive Review. Water Air Soil Pollut..

[6] Ondrasek G., Shepherd J., Rathod S., Dharavath R., Rashid M.I., Brtnicky M., Shahid M.S., Horvatinec J., Rengel Z. (2025). Metal contamination—A global environmental issue: Sources, implications & advances in mitigation. RSC Adv..

[7] Tang S., Wang C., Song J., Ihenetu S.C., Li G. (2024). Advances in Studies on Heavy Metals in Urban Soil: A Bibliometric Analysis. Sustainability.

[8] Bonilla-Bedoya S., López-Ulloa M., Mora-Garcés A., Macedo-Pezzopane J.E., Salazar L., Herrera M.Á. (2021). Urban soils as a spatial indicator of quality for urban socio-ecological systems. J. Environ. Manag..

[9] Rodríguez-Espinosa T., Pérez-Gimeno A., Almendro-Candel M.B., Navarro-Pedreño J. (2024). Constructing Soils to Mitigate Land Occupation by Urban Expansion and Metabolism to Improve Healthy Cities. Land.

[10] Veitch J., Flowers E., Ball K., Deforche B., Timperio A. (2020). Exploring Children’s Views on Important Park Features: A Qualitative Study Using Walk-Along Interviews. Int. J. Environ. Res. Public Health.

[11] Schaffer C.L., White M., Brown C.M. (2018). A Tale of Three Cities: Defining Urban Schools Within the Context of Varied Geographic Areas. Educ. Urban. Soc..

[12] Ribeiro H., de Santana K.V., Oliver S.L. (2024). Natural Environments in University Campuses and Students’ Well-Being. Int. J. Environ. Res. Public Health.

[13] Gkoltsou V.S., Papadimou S.G., Bourliva A., Skilodimou H.D., Golia E.E. (2025). Heavy Metal Levels in Green Areas of the Urban Soil Environment of Larissa City (Central Greece): Health and Sustainable Living Risk Assessment for Adults and Children. Sustainability.

[14] Massas I., Kairis O., Gasparatos D., Ioannou D., Vatougios D., Zafeiriou I. (2023). Impaired Soil Health in Agricultural Areas Close to Fe-Ni Mines on Euboea Island, Greece, Caused by Increased Concentrations of Potentially Toxic Elements, and the Associated Impacts on Human Health. Environments.

[15] Argyraki A., Kelepertzis E., Botsou F., Paraskevopoulou V., Katsikis I., Trigoni M. (2018). Environmental availability of trace elements (Pb, Cd, Zn, Cu) in soil from urban, suburban, rural and mining areas of Attica, Hellas. J. Geochem. Explor..

[16] Bourliva A., Kantiranis N., Papadopoulou L., Aidona E., Christophoridis C., Kollias P., Evgenakis M., Fytianos K. (2018). Seasonal and spatial variations of magnetic susceptibility and potentially toxic elements (PTEs) in road dusts of Thessaloniki city, Greece: A one-year monitoring period. Sci. Total Environ..

[17] Golia E.E., Emmanouil C., Charizani A., Koropouli A., Kungolos A. (2023). Assessment of Cu and Zn contamination and associated human health risks in urban soils from public green spaces in the city of Thessaloniki, Northern Greece. Euro-Mediterr. J. Environ. Integr..

[18] Philandras C.M., Nastos P.T., Paliatsos A.G., Repapis C.C. (2010). Study of the rain intensity in Athens and Thessaloniki, Greece. Adv. Geosci..

[19] Sfetsas T., Ghoghoberidze S., Karnoutsos P., Tziakas V., Karagiovanidis M., Katsantonis D. (2024). Spatial and Temporal Patterns of Trace Element Deposition in Urban Thessaloniki: A Syntrichia Moss Biomonitoring Study. Atmosphere.

[20] Svigkas N., Loupasakis C., Papoutsis I., Kontoes C., Alatza S., Tzampoglou P., Tolomei C., Spachos T. (2020). InSAR Campaign Reveals Ongoing Displacement Trends at High Impact Sites of Thessaloniki and Chalkidiki, Greece. Remote Sens..

[21] Amistadi L., Bradecki T., Uherek-Bradecka B. (2023). Resilient university campus in the city in COVID and post-COVID era—Recommendations, guidelines, and evidence from research in Italy and Poland. Urban Des. Int..

[22] Page A.L. (1982). Front Matter. Methods of Soil Analysis: Part 2 Chemical and Microbiological Properties.

[23] (2005). ISO 10390 Second Edition. www.iso.org.

[24] Bouyoucos G.J. (1962). Hydrometer Method Improved for Making Particle Size Analyses of Soils. Agron. J..

[25] Müller G. (1979). Schwermetalle in den sedimenten des Rheins-Veranderungen seit. Umschav.

[26] Hakanson L. (1980). An ecological risk index for aquatic pollution control.a sedimentological approach. Water Res..

[27] Tomlinson D.L., Wilson J.G., Harris C.R., Jeffrey D.W. (1980). Problems in the assessment of heavy-metal levels in estuaries and the formation of a pollution index. Helgoländer Meeresunters..

[28] Goncharov G., Soktoev B., Farkhutdinov I., Matveenko I. (2024). Heavy metals in urban soil: Contamination levels, spatial distribution and human health risk assessment (the case of Ufa city, Russia). Environ. Res..

[29] El-Sharkawy G., Alotaibi M.O., Zuhair R., Mahmoud E., El Baroudy A., Omara A.E.-D., El-Sharkawy M. (2025). Ecological Assessment of Polluted Soils: Linking Ecological Risks, Soil Quality, and Biota Diversity in Contaminated Soils. Sustainability.

[30] USEPA (2002). Child-Specific Exposure Factors Handbook.

[31] USEPA (1989). Risk Assessment Guidance for Super Fund. Volume I: Human Health Evaluation Manual.

[32] Liu X., Song Q., Tang Y., Li W., Xu J., Wu J., Wang F., Brookes P.C. (2013). Human health risk assessment of heavy metals in soil-vegetable system: A multi-medium analysis. Sci. Total Environ..

[33] USEPA (2007). Guidance for Evaluating the Oral Bioavailability of Metals in Soils for Use in Human Health Risk Assessment.

[34] Ferreira-Baptista L., De Miguel E. (2005). Geochemistry and risk assessment of street dust in Luanda, Angola: A tropical urban environment. Atmos. Environ..

[35] USEPA (2016). Regional Screening Lavels (RSLs) – Generic Tables.

[36] Sager M. (2020). Urban Soils and Road Dust—Civilization Effects and Metal Pollution—A Review. Environments.

[37] Ismayilov A.I., Mamedov A.I., Fujimaki H., Tsunekawa A., Levy G.J. (2021). Soil salinity type effects on the relationship between the electrical conductivity and salt content for 1:5 soil-to-water extract. Sustainability.

[38] Heng T., Ma Y., Ai P., Liu Z., Wu M., Liu C. (2024). The Effects of Soil Salt Stress on the Nitrogen Uptake, Yield Response and Nitrogen Use Efficiency of Cotton in Arid Areas. Agronomy.

[39] Purdy K., Reynolds J.K., Wright I.A. (2024). The Influence of Contamination from Concrete Materials on the Growth and Accumulation of Metals within an Invasive Weed (*Salix* spp.). Water Air Soil Pollut..

[40] Polovina S., Radić B., Ristić R., Kovačević J., Milčanović V., Živanović N. (2021). Soil erosion assessment and prediction in urban landscapes: A new G2 model approach. Appl. Sci..

[41] Rudnick R.L., Gao S. (2003). Composition of the Continental Crust. Treatise on Geochemistry.

[42] Wang C., Hu J., Zhang Y., Di Y., Wu X. (2025). Spatial distribution characteristic, source apportionment, and risk assessment of heavy metals in the soil of an urban riparian zone. Ecotoxicol. Environ. Saf..

[43] Li R., Cai G., Wang J., Ouyang W., Cheng H., Lin C. (2014). Contents and chemical forms of heavy metals in school and roadside topsoils and road-surface dust of Beijing. J. Soils Sediments.

[44] Tassiou S., Kaminari M. (2016). Geochemical Environmental Study of the Urban-Peri urban Area of Volos (Volume A).

[45] Gerouki F., Liakopoulos A. (2016). Geochemical Environmental Study of the Urban Peri-urban Area of Igoumenitsa (Volume A).

[46] Argyraki A., Kelepertzis E. (2014). Urban soil geochemistry in Athens, Greece: The importance of local geology in controlling the distribution of potentially harmful trace elements. Sci. Total Environ..

[47] Papazotos P., Liakopoulos A., Kontodimos K., Koukoulis A. (2024). Integrated geochemical analysis of urban and peri-urban soils: A case study of Lamia City, Greece. Environ. Monit. Assess..

[48] Cicchella D., De Vivo B., Lima A., Albanese S., McGill R.A.R., Parrish R.R. (2008). Heavy metal pollution and Pb isotopes in urban soils of Napoli, Italy. Geochemistry: Exploration, Environment, Analysis.

[49] Giusti L. (2011). Heavy metals in urban soils of Bristol (UK). Initial screening for contaminated land. J. Soils Sediments.

[50] Cachada A., Dias A.C., Pato P., Mieiro C., Rocha-Santos T., Pereira M.E., Da Silva E.F., Duarte A.C. (2013). Major inputs and mobility of potentially toxic elements contamination in urban areas. Environ. Monit. Assess..

[51] Alekseenko V., Alekseenko A. (2014). The abundances of chemical elements in urban soils. J. Geochem. Explor..

[52] Tanner P.A., Ma H.-L., Yu P.K.N. (2008). Fingerprinting Metals in Urban Street Dust of Beijing, Shanghai, and Hong Kong. Environ. Sci. Technol..

[53] Wang M., Zhang H. (2018). Accumulation of heavy metals in roadside soil in urban area and the related impacting factors. Int. J. Environ. Res. Public Health.

[54] Mostafa M.T., El-Nady H., Gomaa R.M., Abdelgawad H.F., Abdelhafiz M.A., Salman S.A.E., Khalifa I.H. (2024). Urban geochemistry of heavy metals in road dust from Cairo megacity, Egypt: Enrichment, sources, contamination, and health risks. Environ. Earth Sci..

[55] Siddig M.M.S., Asabere S.B., Al-Farraj A.S., Brevik E.C., Sauer D. (2025). Pollution and ecological risk assessment of heavy metals in anthropogenically-affected soils of Sudan: A systematic review and meta-analysis. J. Hazard. Mater. Adv..

[56] Shen C., Huang S., Wang M., Wu J., Su J., Lin K., Chen X., He T., Li Y., Sha C. (2024). Source-oriented health risk assessment and priority control factor analysis of heavy metals in urban soil of Shanghai. J. Hazard. Mater..

[57] Li Y.-M., Ma J.-H., Liu D.-X., Sun Y.-L., Chen Y.-F. (2015). [Assessment of heavy metal pollution and potential ecological risks of urban soils in Kaifeng City, China]. Huan Jing Ke Xue.

[58] Aslanidis P.S.C., Golia E.E. (2022). Urban Sustainability at Risk Due to Soil Pollution by Heavy Metals—Case Study: Volos, Greece. Land.

[59] Soltani-Gerdefaramarzi S., Ghasemi M., Ghanbarian B. (2021). Geogenic and anthropogenic sources identification and ecological risk assessment of heavy metals in the urban soil of Yazd, central Iran. PLoS ONE.

[60] McKenzie E.R., Money J.E., Green P.G., Young T.M. (2009). Metals associated with stormwater-relevant brake and tire samples. Sci. Total Environ..

[61] Kelepertzis E., Stathopoulou E. (2013). Availability of geogenic heavy metals in soils of Thiva town (central Greece). Environ. Monit. Assess..

[62] Baran A., Wieczorek J., Mazurek R., Urbański K., Klimkowicz-Pawlas A. (2018). Potential ecological risk assessment and predicting zinc accumulation in soils. Environ. Geochem. Health.

[63] Baltas H., Sirin M., Gökbayrak E., Ozcelik A.E. (2020). A case study on pollution and a human health risk assessment of heavy metals in agricultural soils around Sinop province, Turkey. Chemosphere.

[64] Gopal V., Krishnamurthy R.R., Indhumathi A., Sharon B.T.X., Priya T.S.D., Rathinavel K., Bharath K.M., Magesh N.S., Ayyamperumal R. (2024). Geochemical evaluation, ecological and human health risk assessment of potentially toxic elements in urban soil, Southern India. Environ. Res..

[65] Pan L., Wang Y., Ma J., Hu Y., Su B., Fang G., Wang L., Xiang B. (2018). A review of heavy metal pollution levels and health risk assessment of urban soils in Chinese cities. Environ. Sci. Pollut. Res..

[66] Wise S.S., Aboueissa A.E.-M., Martino J., Wise J.P. (2018). Hexavalent Chromium–Induced Chromosome Instability Drives Permanent and Heritable Numerical and Structural Changes and a DNA Repair–Deficient Phenotype. Cancer Res..

[67] Thompson C.M., Suh M., Proctor D.M., Harris M.A. (2018). Letter to the Editor Regarding Banu et al. (2018). Chromium Accumulation on Human Placental Oxidative Stress and Apoptosis. Toxicol. Sci..

